# Supplementary material for: The impact of the COVID-19 pandemic on stress and coping in parents of children with Autism Spectrum Disorder
Source: PLoS One. 2024 Nov 7;19(11):e0313426. doi: 10.1371/journal.pone.0313426 (PMC11542825; doi:10.1371/journal.pone.0313426)
Supplement: Attachment — Submitted filename: Response to Reviewers.docx [file pone.0313426.s002.docx]

Dear Editor and Reviewers, dear Dr. Bolan,

We sincerely appreciate your insightful and valuable comments on our research article, "The Impact of the COVID-19 Pandemic on Stress and Coping in Parents of Children with Autism Spectrum Disorder," and for the opportunity to submit a revised manuscript.

Your feedback is highly valuable to us and has helped us enhance the quality of our work. We have made every effort to address your constructive comments and suggestions comprehensively.

**Regarding the journal requirements:**

1) We have reviewed the stylistic templates provided by PLOS ONE. We believe that the research article meets the stylistic requirements of the journal, including those concerning file naming.

2-3) We have revised the wording of the *Financial Disclosure* as requested. The current wording is as follows: "This work was funded by the Ministry of Health of the Czech Republic (AZV NU22-D-130). The funders had no role in study design, data collection and analysis, decision to publish, or preparation of the manuscript." Additionally, as per your instructions, we have removed any text related to funding from the manuscript. Kindly incorporate this change in the online submission form, as suggested by yourselves.

4) The full data for the study has been added to the online form in the *Supporting information files* section. It was not possible to include open-ended responses in the published dataset, as they contain identifying information about the subjects and do not include variables that are part of the main analysis.

5) We have added the full names of both ethics committees and the method of obtaining their consent to the relevant section in the *Methods* section.

**Reviewers' comments section:**

1) To ensure that readers fully understand the survey content, we have added the full wording of items (k and l) and items (10, 37, 43, and 45) to the *Measures* section, which were omitted from the final survey due to technical errors.

2) We have reworked the paragraph on *Unavailability of care and mental health services* as recommended. We have provided more detailed results regarding the stress associated with the unavailability of healthcare and mentioned them both in the *Results* section and in the relevant paragraph of the *Discussion*. This better connects the results of our study with the discussion and expands upon them with specific parental concerns from the open-ended responses.

3) We have corrected and completed the inadvertently unfinished sentence between lines 321 to 323.

4) We have chosen to retain the *Study Limitations* section in its unchanged form.

5) Thank you for your comment on the topic of neurodiversity and the corresponding tone of the entire text. We have decided to fully respect this principle; therefore, we have substantially reworked the entire article in line with the Neurodiversity Paradigm advocated by WHO. For example, we replace inappropriate terms like "Child with ASD" with "Autistic child/child with autistic features," "Healthy children" with "Neurotypical children" etc. Instead of stigmatization, we aim to promote awareness, understanding, and acceptance of diversity.

Thank you once again for your thorough review and guidance throughout this process. We hope our revised manuscript will now be acceptable for publication. If you have any further suggestions for revisions, we would be happy to address them.

Yours sincerely,

Pavlina Danhofer and other members of the research team
